# Classification and Discrimination of Different Fungal Diseases of Three Infection Levels on Peaches Using Hyperspectral Reflectance Imaging Analysis

**DOI:** 10.3390/s18041295

**Published:** 2018-04-23

**Authors:** Ye Sun, Kangli Wei, Qiang Liu, Leiqing Pan, Kang Tu

**Affiliations:** College of Food Science and Technology, Nanjing Agricultural University, No. 1, Weigang Road, Nanjing 210095, China; 2015208018@njau.edu.cn (Y.S.); 2016108043@njau.edu.cn (K.W.); 2016208004@njau.edu.cn (Q.L.); pan_leiqing@njau.edu.cn (L.P.)

**Keywords:** hyperspectral imaging, deep learning, postharvest diseases, peaches, decayed levels

## Abstract

Peaches are susceptible to infection from several postharvest diseases. In order to control disease and avoid potential health risks, it is important to identify suitable treatments for each disease type. In this study, the spectral and imaging information from hyperspectral reflectance (400~1000 nm) was used to evaluate and classify three kinds of common peach disease. To reduce the large dimensionality of the hyperspectral imaging, principal component analysis (PCA) was applied to analyse each wavelength image as a whole, and the first principal component was selected to extract the imaging features. A total of 54 parameters were extracted as imaging features for one sample. Three decayed stages (slight, moderate and severe decayed peaches) were considered for classification by deep belief network (DBN) and partial least squares discriminant analysis (PLSDA) in this study. The results showed that the DBN model has better classification results than the classification accuracy of the PLSDA model. The DBN model based on integrated information (494 features) showed the highest classification results for the three diseases, with accuracies of 82.5%, 92.5%, and 100% for slightly-decayed, moderately-decayed and severely-decayed samples, respectively. The successive projections algorithm (SPA) was used to select the optimal features from the integrated information; then, six optimal features were selected from a total of 494 features to establish the simple model. The SPA-PLSDA model showed better results which were more feasible for industrial application. The results showed that the hyperspectral reflectance imaging technique is feasible for detecting different kinds of diseased peaches, especially at the moderately- and severely-decayed levels.

## 1. Introduction

Fresh peach fruits are susceptible to infection by several postharvest pathogens, since they are typically picked in hot and rainy seasons. Their tender texture, appealing flavor, and abundant nutrition result in easy susceptibility to deterioration, and their flavor changes quickly within three to five days at ambient temperatures [1]. Gray mold, soft rot and anthracnose caused by *Botrytis cinerea*, *Rhizopus stolonifera*, and *Colletotrichum acutatum*, respectively, are the major postharvest diseases of peaches [2]. In order to avoid potential health risks, infected peaches must be identified before they are stored, sold or processed. Thus, there is a need for a rapid and reliable technique to detect and differentiate between the pathogens of food or agricultural commodities that may cause severe outbreaks. On the other hand, to control disease, it is important to identify suitable treatments for each disease type.

Currently employed, nondestructive methods for the identification of diseased fruit caused by spoilage fungi mainly rely on techniques such as the computer vision system [3], NIR spectroscopy [4], and the electronic nose device [5]. Although some of these methods are effective for detecting defects, there is insufficient information on whether they can classify different fungal diseases. Conventional methods for classification of different fungi mainly rely, firstly, on specific microbiological and biochemical identification, including the enzyme-linked immunosorbent assay (ELISA) [6], the fluorescence polarization immunoassay [7] and the polymerase chain reaction (PCR) method [8], although these methods are sensitive and give both qualitative and quantitative information about the tested microorganisms, high-skilled operators and precise control of the experimental conditions are commonly required. Secondly, conventional methods rely on morphological characteristics; these methods are fastidious and frequently lead to false negatives because the fungi are often overgrown or infected by other micro-organisms in the media [9]. Some new non-destructive methods have also been applied to classify bacteria. Banada [10] described a light-scattering sensor for construction of a scatter-signature image library that is able to distinguish five species with an accuracy of 90–99% for samples derived from food or experimentally-infected animals. Kumar [11] explored the use of fluorescence microscopy and image analysis for the identification and classification of micro-organisms. Pahlow [12] applied Raman spectroscopy for the detection of bacteria-like sample preparation and the identification process. Nevertheless, the applicability of these methods is somewhat restricted because of the complexity and diversity of biological materials which can easily interfere with images or spectra. Thus, is important that a rapid and non-destructive inspection system is developed which can not only detect the diseased fruit but also differentiate between the different diseases.

Hyperspectral imaging technology combines conventional imaging and spectroscopic techniques to simultaneously acquire imaging and spectral information, which enables us to detect external or internal quality attributes of agricultural product rapidly [13]. Consequently, hyperspectral imaging has attracted the interest of researchers as a new tool for food defect inspection over the past decade [14]. Sun [2] applied hyperspectral imaging to model the growth and discrimination of spoilage fungi infection in real food. Li [15] presented a multispectral detection method for skin defects (including decay and disease spots) of bicolored peaches based on visible–near infrared (vis–NIR) hyperspectral imaging. Sun [16] selected the optimal wavelengths of hyperspectral imaging via chlorophyll content, and then detected fungal diseases in peaches. The hyperspectral imaging technique combined with conventional linear and non-linear machine-learning models, such as the partial least squares (PLS) method, and support vector machine (SVM) have played important roles in classification analysis. With the developing of deep learning, convolutional neural networks (CNN) and deep belief networks (DBN) have been used with high frequency for image classification. Compared with conventional machine-learning technology, deep learning technology shows a higher accuracy rate for object location, signal recognition and human face recognition [17]. In contrast, the application of deep learning technology in the detection of agricultural products is still in its infancy.

Researchers have used visible and near infrared hyperspectral imaging (HIS) to identify rotten fruit, and satisfactory results have been obtained [2,15,16,17]. However, to the best of our knowledge, up until now, there have been no reports regarding discrimination between different postharvest diseases in peaches using HSI. In addition, the time phase of diseased fruit is also very important, so different time phases of decay were also considered in this research. In this paper, one type of deep learning technology—beep delief networks—is introduced into hyperspectral data processing. The results are compared to the classification results of a conventional linear model (partial least squares discriminant analysis, PLSDA), and related to the discrimination of different types of microbial spoilage. The main objectives of this work were to (1) detect the decayed peaches in different periods; (2) classify different fungal diseases using spectral and imaging information, (3) compare the classification performance of a conventional linear model and deep learning technology, and (4) select the optimal information for disease detection.

## 2. Materials and Methods

### 2.1. Fungal Strains and Culture

Fungal stains were selected based on their frequent occurrence during the storage of peaches. *Botrytis cinerea* (*B. cinerea*) and *Rhizopus stolonifera* (*R. stolonifera*) were bought from Guangdong Micrology Culture Center (Guangdong, China), and *Colletotrichum acutatum (C. acutatum)* was supplied by the College of Food Science and Technology at Nanjing Agricultural University of China. The strains were cultured on potato dextrose agar (PDA) plates in an incubator at 28 °C and 85% relative humidity conditions for 7 days. Then, heavily sporulating cultures were obtained. Spores were collected by flooding the plates with sterile, distilled water containing 10 mL/L of Tween 80. After filtering the spore suspension through four layers of sterile filter paper, the final spore concentration was adjusted to 4 × 10^4^ spores/mL by a hemocytometer.

### 2.2. Peach Samples

The peaches, named “Xiahui 5”, were handpicked from the orchard of the Academy of Agricultural Sciences in Jiangsu province. The peaches without defects, bruises, diseases, and contamination were selected and randomly divided into four groups (30 samples for the control group, 270 samples for the three treated groups, 90 samples for each treated group). To sterilize the surface bacteria, the peaches were soaked in sodium hypochlorous acid solution (1%) for two minutes. The peaches were repeatedly cleansed using sterile, distilled water. After the peaches were air-dried at room temperature, the treatment groups were artificially inoculated with the spore suspensions of *R. stolonifera*, *B. cinerea* and *C. acutatum*, respectively, using a syringe with a steel needle. Each sample was injected with about 10 μL of the fungal spore solution to a depth of approximately 5 mm. The treated samples were carefully placed in a thermotank and stored at 20 °C. The decayed spots appeared in peaches 2 days after the spores were cultured. To obtain peaches at different stages of disease, the samples were collected at 24-h intervals for hyperspectral imaging detection and evaluation of the degree of rottenness. After inoculation for four days, the peaches were severely rotted with all three kinds of disease.

### 2.3. Evaluation of Decay Levels

With the growth of fungus, the peaches rotted more and more severely. After about one day of the fungi injection, it was hard to find any decayed spots on most peaches. After four days of inoculation, for most samples, the mycelium had grown out and different symptoms of fungal diseases emerged. However, it is meaningless to detect and classify extremely rotten samples. In this research, we left out the samples which were extremely rotten or had formed obvious hyphae. Because of the individual differences, peaches expressed different sensitivities to fungi—a few peaches were not rotten, while several peaches rotted heavily. Therefore, in this research, the diseased levels of the peaches were grouped by sensory evaluation. Although the development of different fungal diseases varied, the same standards were used to evaluate the stages of decay of peaches; this was based on the average diameter of decayed spots reported by Zhu [18]. The evaluation standards were set according to the diameter (d), and the samples were classified into ‘sound (healthy samples)’, ‘slightly-decayed (0 < d ≤15 mm)’, ‘moderately-decayed (15 < d ≤30 mm)’ and ‘severely-decayed (d >30 mm)’ for each disease.

### 2.4. Hyperspectral Image System and Image Acquisition

Hyperspectral images of peaches were acquired using a lab-scale hyperspectral imaging system in reflection mode, which is similar with the schematic diagram in our previous paper [19]. The system mainly consists of a hyperspectral imaging unit, an adjustable halogen lamp with a range of 0–150 W (3900ER, Illumination Technologies Inc., New York, NY, USA), a horizontal motorized stage (IRCP0076 of-ICOMB001, Isuzu, Taiwan), a computer and image acquisition software (Spectral Image, Isuzu, Taiwan). The hyperspectral imaging unit comprised a CCD camera (ICL-B1620, Imperx, Boca Raton, FL, USA), an imaging spectrometer (ImSpectorV10E, Specim, Oulu, Finland), and variable focal length lens. The parameters of the hyperspectral imaging system were set as the following: the conveyor speed was adjusted to 4 mm/s, the distance between a sample and the camera or a light source was 20 cm, the intensity of the light source was 45 W with a 45° angle to the sample, and the exposure time was 5 ms. The obtained image consisted of 420 spatial profiles with wavelengths ranging from 400 to 1000 nm at intervals of 1.43 nm.

The samples were scanned one by one to obtain the hyperspectral image. The acquired raw images had to be corrected with a white and a dark reference image to reduce the influence of illumination and dark current of the CCD detectors as well as the differences in the camera and the physical configuration of the imaging system. This was done with the following equation:

(1) $$R=\frac{R_{0}-R_{d}}{R_{w}-R_{d}}$$

where *R*_0_ is the original hyperspectral image, *R_d_* is the dark image, and *R_w_* is the white reflectance image. The dark reflection image was obtained by completely covering the camera lens using an opaque cap, and a white reflection image was obtained using a Teflon white board (99% reflectivity) [20]. The corrected images were used for further analysis.

### 2.5. Extraction of Spectral Characteristics

Hyperspectral imaging acquisition generates a large amount of spectral data, and it can collect spectral information for each pixel in the image. At first, each spectrum was extracted from the regions of interest (ROI) using ENVI software (ENVI4.7, Research System Inc., Boulder, CO, USA). To make the ROI of each type of tissue more representative, a circle ROI, varying in size based on the rotten area, was used in the processing. In this study, ‘autoscale’ was used for the pre-processing of the extracted spectrum, using the PLS Toolbox 7.5 (Eigenvector Research, Inc., Wenatchee, WA, USA).

### 2.6. Morphologic Characteristics Extraction

**Figure 1:**
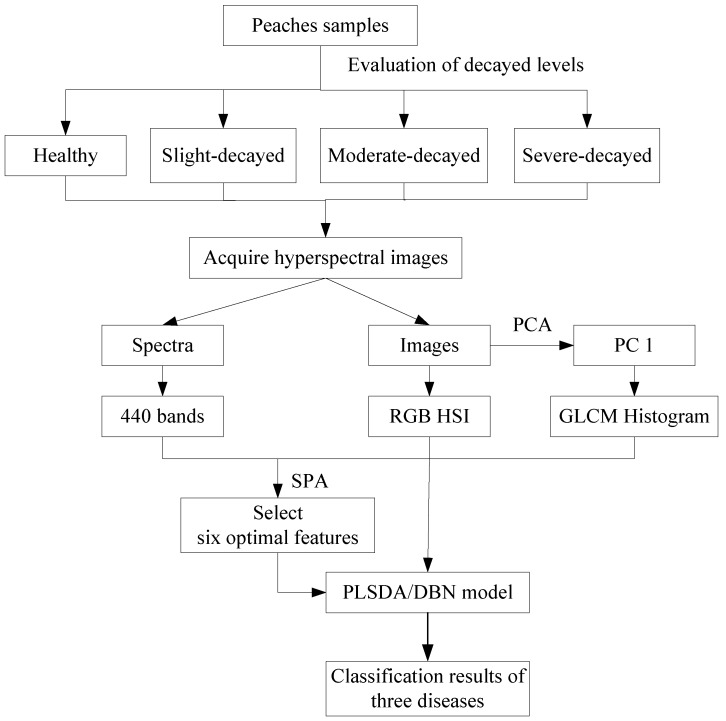
Flowchart of the data analysis procedures used to classify different fungal diseases. (PCA: principal components analysis; PC1: first principal component image; SPA: successive projections algorithm; RGB: red, blue, and green; HIS: hue, saturation, and lightness; GLCM: gray level co-occurrence matrix; PLSDA: partial least squares discrimination analysis; DBN: deep belief network).

The data analysis procedure is shown in Figure 1 Morphologic characteristics, extraction steps, RGB (Red, Green, and Blue) and HSI (Hue, Saturation, Intensity) color spaces were used for describing different aspects of image color. In the RGB space, *I*_R_, *I*_G_, and *I*_B_, refer to the red component, the green component and the blue component, respectively. *I*_H_, *I*_S_, and *I*_I_ refer to the hue component, the saturation component and the lightness component in HSI space, respectively. Considering the amount of computation, simple color was extracted from each image and R, G, B, H, S, and I were the average grey values of the R, G, B, H, S, and I components that were used as the color parameters. The R, G, B, H, S, and I of each type of sample image were calculated using Matlab 2017b.

The gray level co-occurrence matrix (GLCM) is a classic spatial and textural feature extraction method, which is based on the local spatial changes in intensity or color brightness. It is widely used for the texture analysis and pattern recognition of remote sensing data [21]. In most real applications, GLCM textures are usually extracted from a mono-spectral image. However, hyperspectral images have a multi mono-spectral image. If the textural features are calculated for each spectral band, there will be a large amount of redundant and intercorrelated textural information, and it will increase storage requirements and cause computational burden. Consequently, in this study, the first principal component was used to extract the GLCM textures, and the ENVI software package was employed to extract the first principal component image from the full wavelength range with the principal components analysis (PCA) method. Finally, the energy (ENE), contrast (CON), entropy (ENT), and inverse difference (INV) were extracted from four directions (0°, 45°, 90°, 135°) by Matlab 2009a (The Math Works Inc. Natick, MA, USA); therefore, 16 features were extracted from GLCM textures. In addition, the gray histogram, which is also an important feature of an image, can be regarded as the approximate expression of a density function of gray [22]. Histogram counts of 32 bins were extracted from the first principal component image.

Thus, there were total 54 parameters extracted from one sample, 6 color features, 16 from GLCM, and 32 from histogram counts.

### 2.7. Data Processing and Analysis

Figure 1 shows the data analysis procedure. The SPA (successive projections algorithm) was used to determine the key features. In order to discriminate between the decayed peaches and different diseases, the partial least squares discrimination analysis (PLSDA) was used to build a discrimination model by PLS Toolbox 7.5 (Eigenvector Research, Inc., Wenatchee, WA, USA). Spectral characteristics and morphologic characteristics were used in the discrimination model, and then the spectral and morphologic characteristics were combined as the input data for the PLSDA model. The program codes for the deep belief network (DBN) were written using Matlab 2017 (The Mathworks Inc., Natick, MA, USA). DBN can be efficiently trained in an unsupervised, layer-by-layer manner which comprises restricted Boltzmann machines (RBM) [23]. In this study, the DBN model had one input layer, one output layer and two hidden layers. For the four-class classification, each sample was marked as (1,0,0,0)—healthy peaches, (0,1,0,0)—slightly-decayed peaches, (0,0,1,0)—moderately-decayed peaches, or (0,0,0,1)—severely-decayed peaches. The data arrays (spectral characteristics or morphologic characteristics) of the sample were used as the input data, and all input data were converted into a number between 0 and 1 by vector normalization, and the number of array symbols was used as the output data. Then, the predicted number of an array was compared to the actual number of the array to determine the classification accuracy. The DBN model built with the full spectral range had an input layer of 420 units, and the unit number of the first hidden layer and the second hidden layer were 100 and 50 respectively, and the output layer was equal to the number of classification classes. Thus, the structure of the DBN in terms of full spectral information for the four-class classification was 420-200-50-4 in this study. Similarly, the structures of the DBN in regard to image information and combined information for the four-class classification were 54-50-10-4 and 474-200-50-4, respectively. The maximum numbers of training epochs in the RBM training process and the back propagation learning process were respectively set to 5000 and 10,000. Finally, the selected key features were used to establish the SPA-PLSDA and SPA-DBN models. Two thirds of the samples were used as the calibration set, and the remaining samples were used for testing.

## 3. Results and Discussion

### 3.1. Evaluation of Decayed Levels

After two days culturing, most samples inoculated with fungi showed decay damage, with diameters varying between 5 mm and 15 mm, which was still barely visible to the human eye without careful observation, especially the samples inoculated with *C. acutatum*. The early and slight decay was hard to distinguish from the sound peaches, not only because of the small size of the decayed area, but also the small color difference between the sound part and slight decayed part. In addition, slightly decayed peaches had some utilization and economic value. On the third and fourth day after inoculation, with the development of fungus, the decayed area became bigger, and the color of the decayed area turned brown [19]. During the culturing, the disease caused by *R. stolonifera* showed the most serious rottenness, and the diseases caused by *B. cinerea* and *C. acutatum* showed relatively mild symptoms. In this research, the decayed levels of the peaches were grouped by the diameters of the decayed spots. After human inspection, based on the rules shown in Section 2.3, there were three levels for decayed peaches which were inoculated with *R. stolonifera*: 30 samples were ‘slightly-decayed’, 30 samples were ‘moderately-decayed’ and 30 samples were ‘severely-decayed’. Similarly, the grouping results were the same for *B. cinerea* and *C. acutatum*, 30 samples were ‘slightly-decayed’, 30 samples were ‘moderately-decayed’ and 30 samples were ‘severely-decayed’. In addition, 30 healthy peaches were selected for the control group (grouped as ‘sound’).

### 3.2. Spectral Analysis

**Figure 2:**
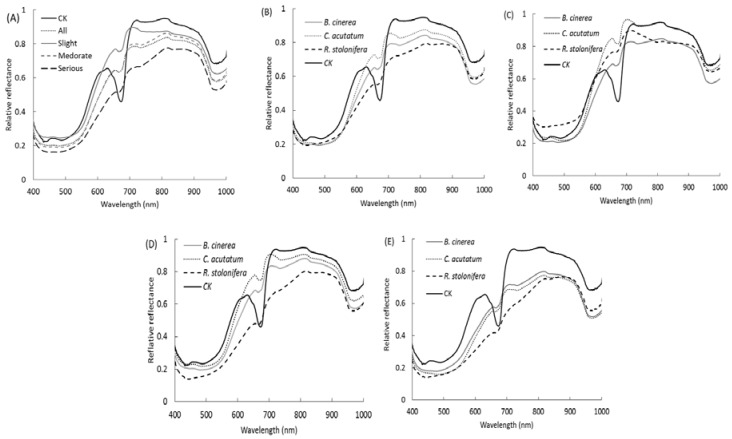
Average reflectance spectra of three kinds of disease and control group using the entire spectral region from 400 to 1000 nm for (**A**) different decay stages of all kinds of diseases, (**B**) different kinds of diseases of all decay stages, (**C**) slightly-decayed samples of different diseases, (**D**) moderately-decayed samples, (**E**) severely-decayed samples.

The typical reflectance spectra of healthy peaches and three kinds of decayed peaches at different levels of decayare shown in Figure 2. Figure 2A shows that the total average spectra of the peaches at different decay stages and the control group (CK), “all” presented the average spectra of total 270 decayed samples. The mean spectra of the three decay stages were calculated based on pixels of the ROIs from all 90 samples (30 for each disease group), and the spectrum of CK was averaged from 30 samples. There was an absorption peak observed at 675 nm of the spectra which was significantly different between decayed and sound peaches. It was reported as the absorption peak of chlorophyll for peaches [24], apples [25], and tomatoes and kiwifruit [26]. There was another absorption peak at around 970 nm of the spectra which was reported as the absorption peak of second overtone O–H stretching from water [27]. Figure 2A shows that the absorption peak of healthy sample was the lowest, and with the development of decay, the absorption intensity increased too. A possible explanation is that the cell structure of the healthy sample is intact, so the state of moisture in the peach can be classified into water and water-free constituents. With the development of decay, the fungi destroy the cell wall and membrane, so the water constituent is released, causing the difference in water content between decayed and sound peaches. The valley was significantly lower in the rotten areas than that of the sound areas, which is possibly explained by the speeding up of synthesized chlorophyll decomposition when the disease occurs [16]. With an increase of decay degree, the differences between decayed and healthy samples increased. This meant that it was easy to classify the diseased samples in the severely-decayed period. According to Figure 2A, we can see that the spectra have obvious differences between diseased and healthy samples which mean we can use the spectra to classify the diseased and healthy samples. After comparing the decayed and healthy samples, discrimination between the three diseases should be considered as the next step. Figure 2B shows the average spectra of each treatment group and control group. The mean spectra of the three diseases were calculated based on pixels of the ROIs from all 90 samples. At wavelengths of 550 to 660 nm and 700 to 810 nm, the reflectance of decayed areas was lower than the reflectance of sound areas. Figure 2C–E shows the hyperspectral reflectance images of ‘slightly-decayed’, ‘moderately-decayed’ and ‘severely-decayed’ samples, respectively. Similarly, there was a large difference at 675 nm between the reflectance spectra of decayed and healthy samples for all three levels. For the slightly-decayed stage (Figure 2C), the spectra overlapped and appeared irregular. It seems to be hard to separate these three kinds of disease at the slightly-decayed stage. As shown in Figure 2D, the spectral values presented a regular pattern to some extent. The reflectance spectrum values of *C. acutatum* were higher than the reflectance spectra values of the other two diseases, while the reflectance spectrum values of *R. stolonifera* were the lowest. These results are consistent with the report by Sun [2], who indicated that *R. stolonifera* grew at the fastest rate among the three fungi. Because of the high growth rate, the decayed area caused by *R. stolonifera* was bigger and turned darker brown; meanwhile, the internal cell wall modifications changed rapidly, both chemically and physically. Consequently, the reflectance decreased faster than the reflectance values of the other two diseases in both the visible and near-infrared (NIR) ranges. Figure 2E indicates that the difference was more significant between the reflectance spectra values of three severely-decayed samples and the control group. For the three diseases, the reflectance of decayed samples had a slow ascending tendency, which was different from the reflectance spectrum tendency of the healthy samples at 700–810 nm. The explanation for this observation was that the values of the absorption coefficient were relatively small, and the light in this wavelength range had a deep penetration which reflected the internal biochemical quality [28].

### 3.3. Image Features Analysis

**Figure 3:**
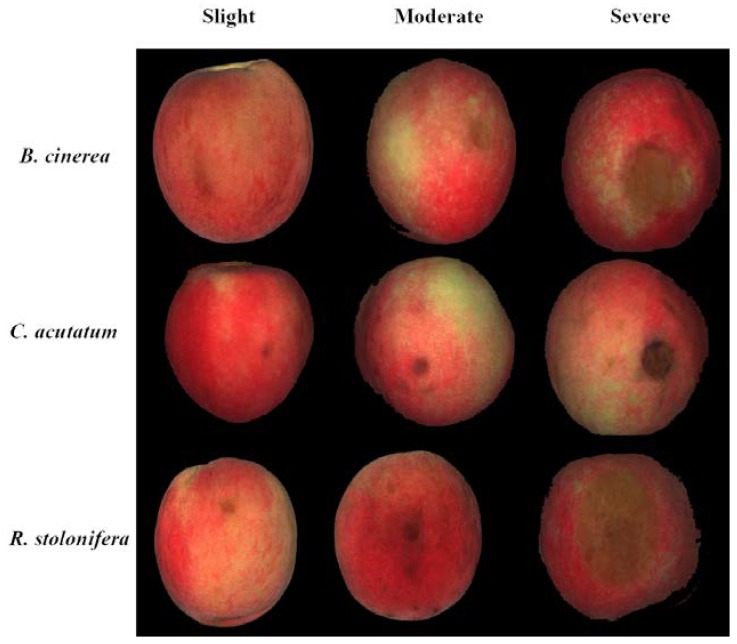
RGB images of the three kinds of diseases at each level of decay.

**Figure 4:**
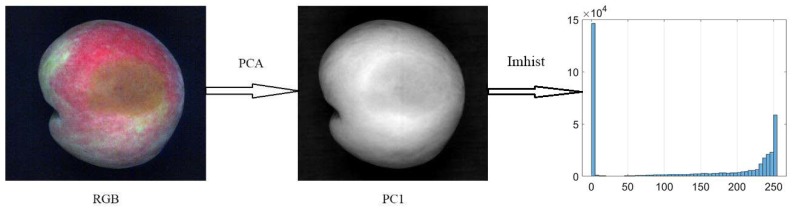
The common procedure for image processing.

Figure 3 shows the RGB (red for 662 nm, green for 554.5 nm, and blue for 450 nm) images of three kinds of decayed peaches at different levels of decay, which were taken from the hyperspectral images. It was hard to distinguish between these three postharvest diseases based on the images, especially at the slightly-decayed level. For the severely-decayed peaches, the diseased spots on *C. acutatum* showed a relatively darker color and a regular circular shape compared to the other two diseases. The basic procedures to extract more image features are shown in Figure 4. In this research, the image of the first principal component (PC) was obtained from the PCA of the hyperspectral reflectance images using all of the spectral channels (400–1000 nm). We found that the first PC image could better maintain the information about the diseased spots; it could also eliminate the influence of the skin color. Then the GLCM textures and grey levels of histogram were extracted from the image of the first principal component.

### 3.4. Discriminant Models for the Classification of Diseased and Healthy Peaches

For the basic classification, the samples were classified as either healthy or diseased. Therefore, for the two-class classification, the diseased peaches were considered as being in the slight, moderate or severe level. The PLS-DA model was used to establish the classification models for diseased and healthy peaches using the full spectral range (420 bands), image information (54 features) and combined information (474 features), as shown in Table 1 (a). The results of all levels showed that the PLSDA model achieves a high classification accuracy for both spectral and image features, especially for the model of spectral information. Compared to the other levels, the slightly-decayed level of the model showed relatively poor results, with accuracies of 91.66% and 88.33% for calibration and prediction based on the combined features. The models based on the combined features had a slight increase in classification accuracy.

Table 1 (b) shows the classification results of the DBN model. The results show that DBN models do help to improve classification accuracy compared to conventional linear models. The best classification accuracy was based on combined features at the severely-decayed level. The results demonstrate that the deep learning network had a strong capacity for complex nonlinear modelling.

The results of the PLSDA and DBN models show that it is easy to detect decayed peaches, and therefore, the classification of the three kinds of disease could be considered as a further step.

### 3.5. Classification of Different Peaches Diseases

The PLSDA algorithms were used to establish the classification models using the full spectral range of 400 to 1000 nm (Table 2 (A-I)). The results show that the classification accuracy of prediction for all levels was almost 85%. The results of all levels represented only classified different fungal diseases and did not consider the levels of decay. The classification accuracy for the peaches with diseases caused by *B. cinerea* and *C. acutatum* was much lower than the classification accuracy for healthy samples. The classification accuracy for healthy samples was almost 100%, which means that a strong spectral difference existed between decayed and healthy peaches. Table 2 (A-II) shows that a poor classification accuracy for *B. cinerea* (40%) was obtained for the slightly-decayed stage—with the development of decay, it was easier to classify the diseases caused by *B. cinerea*. A higher misclassification rate for the “slightly” and “moderately” decayed levels were obtained compared with the low misclassification rate for the “severely” decayed level for the three diseases, which indicated that it was hard to classify these three kinds of diseases at the slightly-decayed stage based on spectra, because of the relatively mild symptoms of the diseases at this decay level. Similar to the modelling method based on the full spectral wavelength range, the modelling results based on image features are shown in Table 2 (B). The model established by the image features had poorer performances compared with the model based on the spectral information. A probable explanation is that the reflectance spectrum was able to reveal more reactions with chemical properties of the fruit [29], so the spectra could better classify the different diseases. Table 2 (B) indicated that healthy peaches can be easily identified. These results are consistent with the classification results based on spectral characteristics. At the severe level of diseased peaches, the classification results were not been as good as the results of the spectral model, which showed that the image feature was limited in the classification application. Table 2 (C) shows that the model based on the integrated information had excellent performance, especially for the slight level, where the accuracy was significantly improved. In general, with adequate information, better classification accuracy can be exhibited, demonstrating that data integration in the current work was feasible for extracting the hyperspectral imaging characteristics. The results in Table 2 showed that the classification of diseases at the slightly-decayed level based on the the conventional linear model was difficult; however, at the moderate and severe levels, although it was hard to classify the three diseases by the naked eye, the spectral and image information could effectively identify them.

The classification accuracy of different diseases using the DBN models built with the spectral and imaging information are shown in Table 3. The results show that DBN models improved the classification accuracy compared to the PLSDA model, especially for at the slightly-decayed level. The classification accuracies using the spectral or imaging information at the severely-decayed level reached 100%. The results of the DBN model demonstrate the possibility for classification of the three fungal diseases in peaches at the slightly-diseased level using a hyperspectral system. In the classification of different diseases, the disease caused by *R. stolonifera* had the highest accuracy rate of the three types of diseases at different levels of decay. The results indicate that the DBN model achieved a higher accuracy rate than the conventional linear model for building classification models for fungal diseases, and would be suitable for complex, high-dimensional and nonlinear analysis of massive data.

### 3.6. Discriminant Models for Decayed Peaches Based on Optimal Features

A total of 300 samples were used to select the effective features from the spectrum and images by SPA (Successive Projections Algorithm). Two-thirds of the samples were used for modelling, and the other one-third of the samples were used for validation. In this research, the effective wavelengths were selected to detect fungal infection in peaches, so the input data contained a total of 300 reflected whole spectra of peaches and decay levels. The contribution of each variable and calculation speed had to be considered to determine the optimal number of variables. According to these factors, the optimal number of variables was selected and RMSECV (Root Mean Square Error of Cross Validation) remained nearly stable after K = 6. Thus, six features were selected by the SPA, five features from the spectrum (688, 711, 810, 910, and 970 nm) and the selected feature was the red component of the color information. The selected features from SPA showed that the spectrum contains more important information than the image—this can also explain the results in Section 3.5 where the discrimination results for the spectral information were better than the discrimination results for the image information. These five optimal wavelengths were in the region of near infrared, and these were because the bands in the near infrared region have deeper penetration than the bands in the visible region [30]. The deeper penetration meant the reflected light could show more information about the chemical properties, physiological statuses and cell structure of the fruit. The wavelength of 688 nm is around the absorption valley of chlorophyll, and this showed a big difference between healthy and diseased peaches. The optimal wavelengths at 711 and 810 nm were the other two reflectance peaks, as shown in Figure 3. The wavelength of 970 nm was ascribed to water content because water is the major component in fruit, as described in Section 3.2 [27]. Since the selected spatial feature was related to the red component, therefore, the band image at 662 nm belonging to the red component of visible region could be selected from the spectra to replace the spatial feature, rather than following a complex data analysis procedure to achieve the red component. Finally, the selected six features were 662, 688, 711, 810, 910, and 970 nm from the spectral information.

The new simplified models were used for classification based on six selected optimal features; this would be more suitable for industrial application. Table 4 (a) shows that the classification accuracies of the PLSDA and DBN models. For the slightly-diseased level, it was hard to differentiate between the three kinds of diseases clearly. However, the diseased samples were not misclassified as healthy samples, which meant that the selected features contain the key information to classify decayed and healthy peaches. The SPA-PLSDA model showed excellent performance for identification of the severely-decayed level, with a 90% classification accuracy in prediction for the four-class classification. The SPA-PLSDA of the moderately-decayed level had a slight decrease in accuracy compared to the severely-decayed level.

The DBN model showed really bad results when the effective features were used. The highest classification accuracy was only 78.3% for the severely-decayed level. A possible explanation might be that the input layer was too small to train a suitable model based on such low dimensional information. Although DBN showed a good performance in the classification of different fungal diseases based on spectral and imaging information, this research shows that it was not suitable for a small number of data. Similar results have been reported by Yang [23]—when the deep learning model was applied to fewer samples in each category in a multi-class classification, this led to overfitting, and conventional machine-learning technologies had certain advantages in this regard.

The results of PLSDA model agreed with the work reported by Sun [31], who used SPA combined with several models to detect chilling injury in peaches by hyperspectral imaging. Their SPA model predicted a poorer result than the original model using full bands. These were because the optimum features contained less information than the full information. The classification results (77.6%, 74.2% for calibration and prediction) of slightly-decayed level peaches from the SPA-PLSDA model in the current study showed that it was hard to classify the three kinds of diseases at early infection whether based on spectral or image information. However, the SPA-PLSDA model showed relative good results in classifying the three diseases at moderate and severe levels using only si features. In this study, the SPA-PLSDA model constructed from fewer variables and minimum redundant features achieved similar prediction results, which indicates that the SPA method selected several effective wavelengths to replace the whole wavelength spectrum (i.e., 6 vs. 494). In general, simplified models with several variables can exhibit similar classification accuracy to the corresponding original models; thus, the selected features in the current work were effective, and the SPA models were feasible for industrial on-line application for the classification of different diseases in peaches. Based on the six selected features, a multi-spectral system could be developed, and the sorting speed of PLS-DA would be higher than the DBN model, which might be more suitable for industrial application.

## 4. Conclusions

The development of hyperspectral imaging systems for non-invasive discrimination of three kinds of diseased peaches was investigated. Three decay levels were used for classification of the diseased peaches. To reduce the large dimensionality of the hyperspectral imaging, principal component analysis (PCA) was performed based on full wavelength imaging. GLCM textures and gray level information were extracted from the first set of PC images. Both the partial least squares discriminant analysis (PLS-DA) model and deep belief network (DBN) model achieved relatively good results for detecting the diseased peaches. For the classification of the three diseases, the DBN model showed the highest classification accuracy of 100% at the severely-decayed stage, which was better than the PLSDA model that was based on combined information from the spectrum and image. The hyperspectral imaging technique did not give satisfactory classification results when the peaches were classified at the slightly-decayed level, because of the mild symptoms on the peach. Six optimal features were selected based on the successive projections algorithm (SPA) from a total of 494 features (662, 688, 711, 810, 910, and 970 nm). The SPA-PLSDA models showed better results than the DBN model which were more feasible for industrial application. The work demonstrates that hyperspectral imaging has the potential to classify different kinds of diseased peaches.

## Figures and Tables

**Table 1:** The results for the classification of diseased and healthy peaches under three levels of decay, using spectral and image features by PLS-DA and DBN models.

Models	Levels	Spectral Features		Image Features		Combined Features	
		Calibration	Prediction	Calibration	Prediction	Calibration	Prediction
(a) PLS-DA	All	99.4	98.8	97.2	95.5	99.7	99.4
	Slight	90.8	86.6	86.6	85	91.6	88.3
	Moderate	100	100	96.6	95	100	100
	Severe	100	100	100	100	100	100
(b) DBN	All	98.7	98.7	97.8	96	99.1	98.7
	Slight	93.3	92	91.56	90.7	95.6	93.3
	Moderate	100	100	100	100	100	100
	Severe	100	100	100	100	100	100

“All” represents samples from all three decay levels.

**Table 2:** The results of the classification of three fungal diseases in peaches for three levels of decay, using the spectral and image features of DBN models.

Level	Classes	(A) Spectral Features		(B) Image Features		(C) Combined Features	
		Calibration	Prediction	Calibration	Prediction	Calibration	Prediction
(I) All	*B. c*	73.3	70	86.6	83.3	88.2	83.3
	*C. a*	93.3	86.6	83.3	66.6	92.5	63.3
	*R. s*	96.6	90	90	76.6	94.1	93.3
	Healthy	100	90	100	100	100	100
	Overall	90.8	84.1	90	81.6	93.7	85.8
(II) Slight	*B. c*	55	40	100	60	56.6	60
	*C. a*	95	80	75	40	95	80
	*R. s*	65	70	80	90	100	93.3
	Healthy	95	100	100	90	100	100
	Overall	77.5	72.5	88.7	70	87.92	83.3
(III) Moderate	*B. c*	70	70	100	90	78.3	90
	*C. a*	90	80	75	70	86.6	90
	*R. s*	100	100	90	60	100	80
	Healthy	100	100	100	100	100	100
	Overall	90	87.5	91.2	80	91.2	90
(IV) Severe	*B. c*	85	90	100	100	100	80
	*C. a*	85	80	100	80	86.6	90
	*R. s*	100	90	95	70	100	100
	Healthy	100	100	100	100	100	100
	Overall	92.5	90	98.7	87.5	96.6	95

*B. c*, *C. a* and *R. s* are the abbreviations of *B. cinerea*, *C. acutatum* and *R. stolonifera*, respectively.

**Table 3:** The results of the classification of three fungal diseases in peaches for three levels of decay, using the spectral and image features of PLS-DA models.

Level	Classes	(A) Spectral Features		(B) Image Features		(C) Combined Features	
		Calibration	Prediction	Calibration	Prediction	Calibration	Prediction
(I) All	*B. c*	83.3	80	86.7	83.3	88.3	85
	*C. a*	93.3	86.6	88.3	83.3	93.3	90
	*R. s*	96.7	93.3	90	86.7	96.7	96.7
	Healthy	100	100	100	100	100	100
	Overall	93.3	90	91.25	88.3	94.6	92.9
(II) Slight	*B. c*	71.7	70	73.3	66.7	80	76.7
	*C. a*	86.7	83.3	76.7	56.7	88.3	85
	*R. s*	80	66.7	80	75	80	73.3
	Healthy	90	90	100	90	100	100
	Overall	82.1	77.5	82.5	72.1	87.1	83.6
(III) Moderate	*B. c*	80	76.7	95	90	91.7	86.7
	*C. a*	93.3	90	90	86.7	95	90
	*R. s*	100	100	100	90	100	100
	Healthy	100	100	100	100	100	100
	Overall	93.3	91.7	96.3	91.7	96.7	94.2
(IV) Severe	*B. c*	100	100	100	100	100	100
	*C. a*	100	100	100	100	100	100
	*R. s*	100	100	100	100	100	100
	Healthy	100	100	100	100	100	100
	Overall	100	100	100	100	100	100

*B. c*, *C. a* and *R. s* are the abbreviations of *B. cinerea*, *C. acutatum* and *R. stolonifera*, respectively.

**Table 4:** Classification results of three fungal diseases in peaches by PLS-DA and DBN models at different decay levels based on optimal spectral and image characteristics.

Model	Classes	All		Slight		Moderate		Severe	
		Cal	Pre	Cal	Pre	Cal	Pre	Cal	Pre
(a) PLS-DA	*B. c*	66.2	63.3	60.8	56.7	68.3	61.7	78.3	76.7
	*C. a*	71.6	59.1	66.2	60	80	81.7	86.7	80
	*R. s*	89.7	86.3	83.3	80	100	80	100	100
	Healthy	100	100	100	100	100	100	100	100
	Overall	81.9	77.2	77.6	74.2	87.1	80.9	91.3	89.2
(b) DBN	*B. c*	58.2	55	50	45.2	56	53.3	71.7	63.3
	*C. a*	70	64.5	63.3	60	66	63.3	76.7	66.7
	*R. s*	72.5	68.8	66.7	63.3	76.7	70	78.3	70
	Healthy	66.5	60	55	50.2	68.3	66.7	68.3	60
	Overall	66.8	62.1	58.8	54.7	66.8	63.3	73.8	65

*B. c*, *C. a* and *R. s* are the abbreviations of *B. cinerea, C. acutatum* and *R. stolonifera*, respectively. Cal and Pre are the abbreviations of the calibration set and prediction set.

## References

[1] Zhang H., Zheng X., Yu T. (2007). Biological control of postharvest diseases of peach with *Cryptococcus laurentii*. Food Control.

[2] Sun Y., Gu X., Wang Z., Huang Y., Wei Y., Zhang M., Tu K., Pan L. (2015). Growth Simulation and Discrimination of *Botrytis cinerea*, *Rhizopus Stolonifer* and *Colletotrichum acutatum* Using Hyperspectral Reflectance Imaging. PLoS ONE.

[3] Li Q., Wang M., Gu W. (2002). Computer vision based system for apple surface defect detection. Comput. Electron. Agric..

[4] Moscetti R., Haff R.P., Saranwong S., Monarca D., Cecchini M., Massantini R. (2014). Nondestructive detection of insect infested chestnuts based on NIR spectroscopy. Postharvest Biol. Technol..

[5] Aparicio R., Rocha S.M., Delgadillo I., Morales M.T. (2000). Detection of Rancid Defect in Virgin Olive Oil by the Electronic Nose. J. Agric. Food Chem..

[6] Muthomi J.W., Ndung’u J.K., Gathumbi J.K., Mutitu E.W., Wagacha J.M. (2008). The occurrence of Fusarium species and mycotoxins in Kenyan wheat. Crop Prot..

[7] Chun H.S., Choi E.H., Chang H., Choi S., Eremin S.A. (2009). A fluorescence polarization immunoassay for the detection of zearalenone in corn. Anal. Chim. Acta.

[8] Farajzadeh Sheikh A., Ahmadi K., Nikakhlagh S. (2016). Detection of Streptococcus pneumoniae and Moraxella catarrhalis in patients with paranasal chronic sinusitis by polymerase chain reaction method. J. Chin. Med. Assoc..

[9] Aroca A., Raposo R. (2007). PCR-Based Strategy to Detect and Identify Species of Phaeoacremonium Causing Grapevine Diseases. Appl. Environ. Microbil..

[10] Banada P.P., Huff K., Bae E., Rajwa B., Aroonnual A., Bayraktar B., Adil A., Robinson J.P., Hirleman E.D., Bhunia A.K. (2009). Label-free detection of multiple bacterial pathogens using light-scattering sensor. Biosens. Bioelectron..

[11] Kumar S., Mittal G.S. (2008). Geometric and optical characteristics of five microorganisms for rapid detection using image processing. Biosyst. Eng..

[12] Pahlow S., Meisel S., Cialla-May D., Weber K., Rösch P., Popp J. (2015). Isolation and identification of bacteria by means of Raman spectroscopy. Adv. Drug Deliv. Rev..

[13] Cen H., Lu R., Ariana D.P., Mendoza F. (2014). Hyperspectral Imaging-Based Classification and Wavebands Selection for Internal Defect Detection of Pickling Cucumbers. Food Bioprocess Technol..

[14] Li J., Rao X., Ying Y. (2011). Detection of common defects on oranges using hyperspectral reflectance imaging. Comput. Electron. Agric..

[15] Li J., Chen L., Huang W., Wang Q., Zhang B., Tian X., Fan S., Li B. (2016). Multispectral detection of skin defects of bi-colored peaches based on vis–NIR hyperspectral imaging. Postharvest Biol. Technol..

[16] Sun Y., Wang Y., Xiao H., Gu X., Pan L., Tu K. (2017). Hyperspectral imaging detection of decayed honey peaches based on their chlorophyll content. Food Chem..

[17] Sun K., Wang Z., Tu K., Wang S., Pan L. (2016). Recognition of Mould Colony on Unhulled Paddy Based on Computer Vision using Conventional Machine-learning and Deep Learning Techniques. Sci. Rep..

[18] Zhang W., Pan L., Zhao X., Tu K. (2016). A Study on Soluble Solids Content Assessment Using Electronic Nose: Persimmon Fruit Picked on Different Dates. Int. J. Food Prop..

[19] Sun Y., Xiao H., Tu S., Sun K., Pan L., Tu K. (2018). Detecting decayed peach using a rotating hyperspectral imaging testbed. LWT Food Sci. Technol..

[20] Zhang W., Pan L., Tu K., Zhang Q., Liu M. (2014). Comparison of Spectral and Image Morphological Analysis for Egg Early Hatching Property Detection Based on Hyperspectral Imaging. PLoS ONE.

[21] Zhang Y. (1999). Optimisation of building detection in satellite images by combining multispectral classification and texture filtering. ISPRS J. Photogramm. Remote Sens..

[22] Lv X., Bai C., Qiu G., Hu M., Zhang S. (2008). Relationship between Mineragraphy Features of Sinter Ore and Its Gray Histogram. ISIJ Int..

[23] Yang H., Hu B., Pan X., Yan S., Feng Y., Zhang X., Yin L., Hu C. (2017). Deep belief network-based drug identification using near infrared spectroscopy. J. Innov. Opt. Health Sci..

[24] Pan L., Zhang Q., Zhang W., Sun Y., Hu P., Tu K. (2016). Detection of cold injury in peaches by hyperspectral reflectance imaging and artificial neural network. Food Chem..

[25] Merzlyak M.N., Solovchenko A.E., Gitelson A.A. (2003). Reflectance spectral features and non-destructive estimation of chlorophyll, carotenoid and anthocyanin content in apple fruit. Postharvest Biol. Technol..

[26] Cubeddu R., D’Andrea C., Pifferi A., Taroni P., Torricelli A., Valentini G., Dover C., Johnson D., Ruiz-Altisent M., Valero C. (2001). Nondestructive quantification of chemical and physical properties of fruits by time-resolved reflectance spectroscopy in the wavelength range 650–1000 nm. Appl. Opt..

[27] Howard D.L., Kjaergaard H.G. (2006). Influence of intramolecular hydrogen bond strength on OH-stretching overtones. J. Phys. Chem. A.

[28] Cen H., Lu R., Mendoza F.A., Ariana D.P. (2012). Assessing Multiple Quality Attributes of Peaches Using Optical Absorption and Scattering Properties. Trans. ASABE.

[29] Bochereau L., Bourgine P., Palagos B. (1992). A method for prediction by combining data analysis and neural networks: Application to prediction of apple quality using near infra-red spectraag. J. Agric. Eng. Res..

[30] Lu R. (2016). Light Scattering Technology for Food Property, Quality and Safety Assessment.

[31] Sun Y., Gu X., Sun K., Hu H., Xu M., Wang Z., Tu K., Pan L. (2017). Hyperspectral reflectance imaging combined with chemometrics and successive projections algorithm for chilling injury classification in peaches. LWT Food Sci. Technol..

